# Theoretical Investigation on the “ON-OFF” Mechanism of a Fluorescent Probe for Thiophenols: Photoinduced Electron Transfer and Intramolecular Charge Transfer

**DOI:** 10.3390/molecules28196921

**Published:** 2023-10-03

**Authors:** Yuxi Wang, Meng Zhang, Wenzhi Li, Yi Wang, Panwang Zhou

**Affiliations:** 1 School of Biological Engineering, Dalian Polytechnic University, Dalian 116034, China; yx.wang@mail.sdu.edu.cn (Y.W.); 15041271707@163.com (M.Z.); 202217024@mail.sdu.edu.cn (W.L.); 2 Institute of Molecular Sciences and Engineering, Institute of Frontier and Interdisciplinary Science, Shandong University, Qingdao 266237, China

**Keywords:** ESIPT, d-PET, thiophenol, frontier molecular orbital

## Abstract

In this study, the sensing mechanism of (2E,4E)-5-(4-(dimethylamino)phenyl)-1-(2-(2,4dinitrophenoxy)phenyl)penta-2,4-dien-1-one (DAPH-DNP) towards thiophenols was investigated by density functional theory (DFT) and time-dependent DFT (TD-DFT). The DNP group plays an important role in charge transfer excitation. Due to the typical donor-excited photo-induced electron transfer (d-PET) process, DAPH-DNP has fluorescence quenching behavior. After the thiolysis reaction between DAPH-DNP and thiophenol, the hydroxyl group is released, and DAPH is generated with the reaction showing strong fluorescence. The fluorescence enhancement of DAPH is not caused by an excited-state intramolecular proton transfer (ESIPT) process. The potential energy curves (PECs) show that DAPH-keto is less stable than DAPH-enol. The frontier molecular orbitals (FMOs) of DAPH show that the excitation process is accompanied by intramolecular charger transfer (ICT), and the corresponding character of DAPH was further confirmed by hole-electron and interfragment charge transfer (IFCT) analysis methods. Above all, the sensing mechanism of the turn-on type probe DAPH-DNP towards thiophenol is based on the PET mechanism.

## 1. Introduction

Thiophenols (PhSH) are colorless liquids with a foul odor. Due to the characteristics of the S–H groups on the benzene rings [1,2], they are often utilized as intermediates in the manufacture of fine chemicals such as colors, pharmaceuticals, insecticides, polymer inhibitors, and antioxidants. Thiophenols are highly toxic and corrosive, when inhaled or ingested by the human body, they can lead to larynx, bronchospasm, edema, chemical pneumonia, and even death. The Environmental Protection Agency has identified thiophenols as priority contaminants. In recent years, the detection of thiophenols in biological and environmental samples has aroused great interest among scholars [3,4,5,6,7].

Traditional methods for detecting thiophenols include inductively coupled plasma mass spectrometry (ICP-MS), electrochemistry, and high-performance liquid chromatography (HPLC) [8,9]. Due to limitations such as poor coordination and low sensitivity, it is difficult to detect appropriately the concentration of thiophenols in vitro and in vivo. Recently, near-infrared (NIR) fluorescent probes for the detection of thiophenols in water samples and living cells have been reported, which have simple synthesis routes, high selectivity, and high sensitivity [10,11,12,13]. Fluorescent probes can detect trace components by observable wavelength changes and fluorescence intensities. Compared to other methods, fluorescent probes can penetrate deep into the tissue, reducing the interference of the autofluorescence of samples, and thus realizing the detection of biological samples. Fluorescent probes have been synthesized primarily by relying on a diverse array of sensing mechanisms, including excited-state intramolecular proton transfer (ESIPT) [14,15,16], chelation-enhanced fluorescence (CHEF) [17,18,19], photoinduced electron transfer (PET) [20,21,22], intramolecular charge transfer (ICT) [23,24], and aggregation-induced enhanced emission (AIEE) [25,26]. ESIPT based fluorescent probes exhibit unique excited-state photophysical properties of high fluorescence quantum yield and large Stokes shift. Fluorescence emission peaks of ESIPT molecules are often red-shifted by tens to hundreds of nanometers compared to their absorption peaks, and their fluorescence spectra have narrow peak widths, resulting in high optical resolution and detection sensitivity. ESIPT is a completely reversible cyclic process. Upon photoexcitation, the H atom of the hydrogen bond H–X…Y is transferred from the electronegative atom X to another electronegative atom Y, accompanied by the generation of a new hydrogen bond (H…X–Y), which is a typical enol–keto tautomerization. As a result, they have broad applications in fields such as biological imaging and drug screening [27,28,29].

**Scheme 1:**
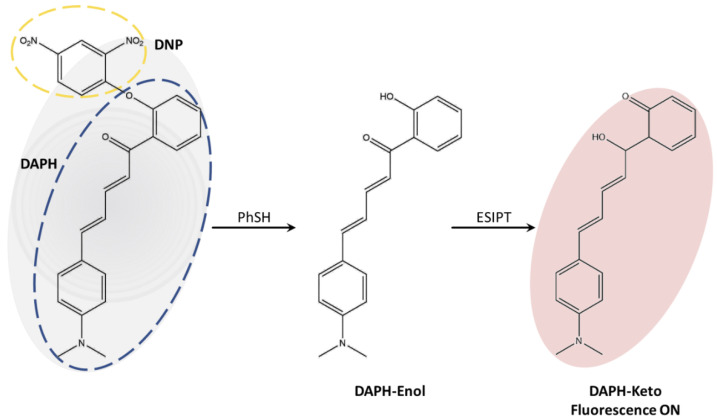
Sensing mechanism of probe DAPH-DNP towards thiophenols.

Recently, a simple NIR-emitting fluorescent probe (DAPH-DNP), consisting of a thiophenol recognition unit (2,4-dinitrophenyl (DNP) group) and a fluorophore (2E,4E)5-(4-(dimethylamino)phenyl)-1-(2-hydroxyphenyl)penta-2,4dien-1-one (DAPH), with large Stokes shift based on the ESIPT mechanism was presented by Li et al. [30], which could be applied to detect effectively thiophenol in water samples and living cells with a remarkable recovery rate (see Scheme 1). Because the DNP group is highly selective to thiophene, the fluorescence of the probe DAPH-DNP is quenched, which hinders the ESIPT process. The nucleophilic substitution process of dinitrophenyl ethers involved in thiophenol release the hydroxyl group and thus restore the ESIPT process, resulting in fluorescence emission (Scheme 1). However, the fluorescence quenching mechanism of DAPH-DNP and the reason for the fluorescence emission of DAPH were not discussed in detail.

In this paper, a theoretical calculation approach was employed to provide a comprehensive account of the fluorescence quenching process of the probe. The potential energy curves were established in order to analyze the ESIPT process. To further investigate the photophysical characteristics and sensing mechanism of DAPH-DNP and DAPH, the charge transfer process was studied utilizing hole-electron [31,32] and IFCT [33] analysis methods.

## 2. Results and Discussion

### 2.1. DAPH-DNP

We first computed the spectral data with different functionals, and the results are listed in Table S1, which indicate that the calculated absorption and emission energies of DAPH-Enol at the B3LYP-D3(BJ) [34,35] level (2.49 eV/2.14 eV) are more comparable to the experimental values (2.68 eV/1.90 eV). However, it is widely recognized that the conventional functional B3LYP might tend to underestimate the energy of the charge transfer (CT) state. On the other hand, the inclusion of solvent effects within the linear-response (LR) scheme may lead to an overestimation of the CT state energy. The interplay between these factors often results in an error-cancellation phenomenon in LR-B3LYP, effectively describing the energy of the CT state [36,37]. By combining a range-separated functional with a corrected LR (cLR) solvation scheme, it is possible to achieve a genuinely accurate depiction of the CT state’s energy. Therefore, we performed the geometry optimizations at the LR-B3LYP level, and then recalculated the energy landscape using the cLR-ωB97XD approach.

**Figure 1:**
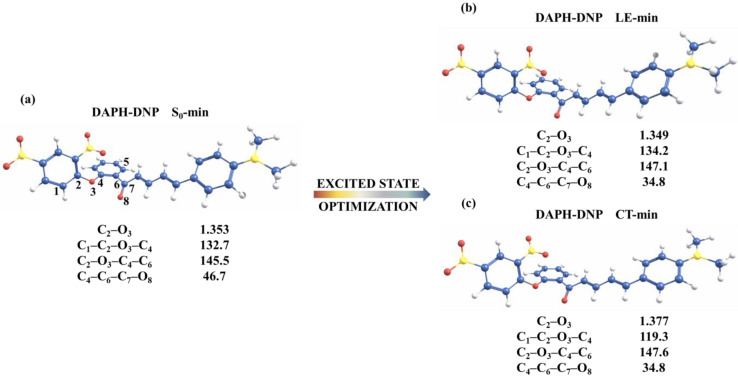
The optimized structures of DAPH-DNP in the S_0_ (**a**), LE (S_1_) (**b**), and CT (S_2_) states (**c**). The labeling of atomic color: O: red; C: blue; H: white; N: yellow, the bond length in Å, the dihedral angle in degrees.

Figure 1a shows the optimized structure of DAPH-DNP in the ground (S_0_-min) state, and the major atoms are marked with numbers. The key geometric parameters of DAPH-DNP are also marked (Figure 1). In the S_0_ state, the DNP group on the benzene ring is not parallel to the fluorophore (DAPH). The plane of the DNP group is approximately 145.5° angle from the plane of the DAPH. As shown in Table 1, the excitation process is dominated by the S_0_ → S_1_, corresponding to the electron transition from the highest occupied molecular orbital (HOMO) to the lowest unoccupied molecular orbital+2 (LUMO+2), and the oscillator strength is 1.8031, which indicates that the first (S_1_) excited state is a bright state. The near-zero oscillator strength in S_0_ → S_2_ implies that the second (S_2_) excited state of DAPH-DNP is a dark state, which corresponds to the electron transition from HOMO-4 → LUMO+2. The key bond lengths and dihedral angles of DAPH-DNP in the S_1_ (LE-min) and S_2_ (CT-min) states are listed in Figure 1b,c. The parameters of the optimized S_1_-state geometry are similar to those in the S_0_ state. The main difference is that the dihedral angle of ∠C_4_C_6_C_7_O_8_ is reduced from 46.7° to 34.8°, which means that the flatness of the DAPH-DNP increases after reaching the S_1_ state. Compared with the S_0_ state, the structural changes in the S_1_ state are mainly manifested by the slight torsion of the DNP group. Unlike the S_1_ state, the dihedral angle of ∠C_1_C_2_O_3_C_4_ in the S_2_ state changes from 132.7° to 119.3°, and the C_2_–O_3_ bond length is increased from 1.353 Å to 1.377 Å. This indicates that the fluorescence quenching behavior of DAPH-DNP may be affected by the DNP group on the benzene ring.

**Figure 2:**
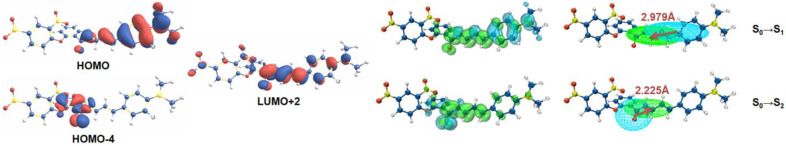
Excitation processes of DAPH-DNP. Molecular orbitals are given in blue and red iso-surfaces, holes and electrons are given in blue and green iso-surfaces, respectively (ωB97XD/TZVP/IEFPCM).

**Figure 3:**
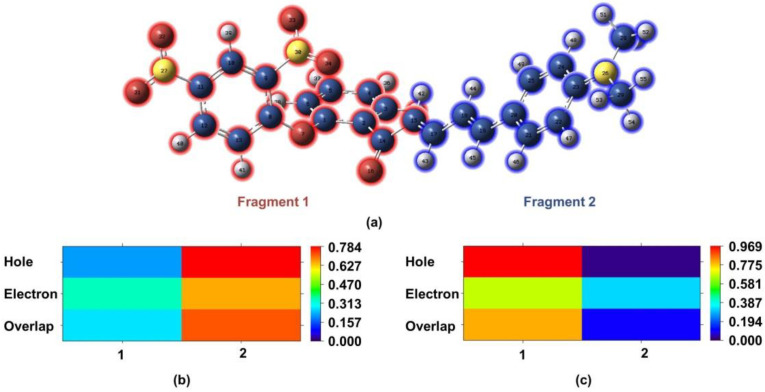
The IFCT analyzing the electron excitation process of DAPH-DNP molecular fragments. (**a**) The amount of electron transfer between fragments from S_0_ to S_1_ (**b**) and S_2_ states (**c**) (ωB97XD/TZVP/IEFPCM).

The plotted FMOs (shown in Figure 2) show that no significant change in electron distribution is observed during the HOMO → LUMO+2 while a substantial charge separation occurs in the HOMO-4 → LUMO+2 transition. Thus, it can be preliminarily judged that the S_1_ and S_2_ states are the LE state and the CT state, respectively. To describe more accurately the characteristics of electron excitation, hole-electron analysis is used. For S_0_ → S_1_, there is incomplete separation of the distribution regions of holes and electrons (corresponding to the location of center of the blue and green iso-surfaces), the centers of the blue and green iso-surfaces are slightly farther apart, the D-index is 2.979 Å, and the t-index is negative (−0.122), indicating a small degree of charge transfer within the fluorophore. For S_0_ → S_2_, there is an obvious charge separation in which holes and electrons are mainly distributed in the *o*-hydroxyacetophenone group and DAPH group, respectively. Moreover, the centers of the hole and electron are relatively far apart, indicating that a significant charge transfer has occurred during the excitation process. As listed in Table 2, the D index of S_0_ → S_2_ excitation is as large as 2.225, the Sr index is very small (0.384), and the t index is 0.201, significantly larger than 0. Therefore, the S_0_ → S_2_ are charge transfer excitations in the *o*-hydroxyacetophenone group in the *N*,*N*-dimethylamino direction. Meanwhile, we also used the IFCT method to quantitatively describe the contribution of each segment of DAPH-DNP to charge transfer. The detailed information is shown in Table S2 and Figure 3. DAPH-DNP is divided into two fragments (Figure 3a). The calculated results show that the contribution of fragment 2 in the S_1_ state to electrons is as high as 96.87%, and differences in the electron transfer of fragment 1 (−12.84%) and fragment 2 (12.84%) indicate that only a small amount of electron transfer from fragment 2 to fragment 1 during S_0_ → S_1_ excitation occurs. Figure 3c shows that the fragment in the S_2_ state has the highest contribution to holes (96.87%), which can be seen from the difference of charge transfer (Table S2) in that the electrons of fragment 1 are significantly reduced (−29.55%), while the electrons of fragment 2 are significantly increased. This means that a significant intramolecular charge transfer during S_0_ → S_2_ excitation is shown. The calculated vertical excitation energy (VEE) in the S_1_ state is 3.14 eV (395 nm) (Table 1), which slightly overestimates the experimental date (2.77 eV, 447 nm).

**Figure 4:**
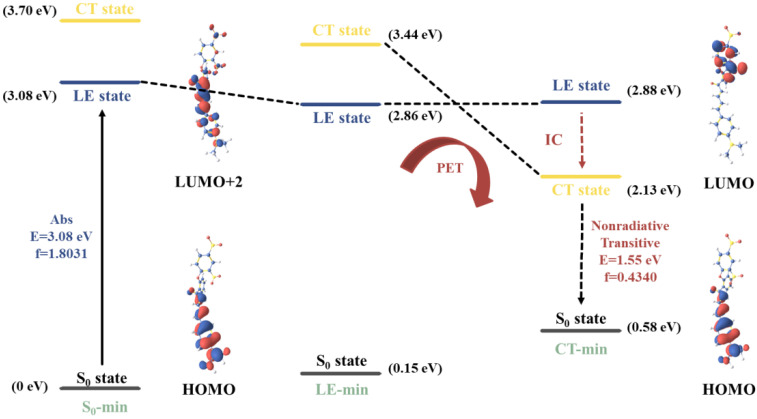
The ωB97XD/TZVP/IEFPCM calculated energies of DAPH-DNP showing the PET mechanism. The red bars: S_2_ state.

To further explain the fluorescence quenching mechanism of the probe, the calculated energy diagrams involved in the excitation and emission processes are plotted. As indicated in Figure 4, at both of the FC point and LE-min, the S_1_ and S_2_ states of DAPH-DNP are in LE and CT states, respectively, and the energy gaps between these two states (CT and LE state) are 0.62 eV and 0.58 eV, respectively. However, at the CT-min, the order of the CT and LE states is reversed, with the S_1_ state transitioning into a CT state. This suggests that the fluorescence quenching mechanism at the cLR-ωB97XD level involves a transformation from the LE to the CT state through a nearly barrierless minimal energy conical intersection (MECI). This mechanism, as reported in previous studies [38,39], aligns well with experimental observations. According to Kasha’s rule [40], the fluorescence emitted by a molecule can only be excited from the S_1_ state. Since the S_1_ state has become a dark CT state at CT-min, this can only return to the ground state by non-radiative decay and is in accordance with the experimentally observed fluorescence quenching behavior of the probe. Briefly, the significant charge transfer from the DAPH unit to the DNP group in relaxation is well in line with the definition of the typical d-PET (donor-excited PET) [41,42].

Additionally, we conducted single point energy calculations using the COSMO-ADC(2) method within the perturbation theory on the energy and density (PTED) reaction field scheme as implemented in Turbomole [43]. The COSMO-ADC(2)/PTED results (Table S3) exhibit a similar energy landscape to that of the cLR-ωB97XD. Moreover, the computed vertical excitation energy is in closer agreement with the experimentally measured absorption maximum compared to the cLR-ωB97XD. However, at the CT-min, the computed energy gap between the ground state and the CT state is only 0.08 eV, suggesting that the COSMO-ADC(2)/PTED might underestimate the energy of the CT state, in line with previous benchmark studies [44].

### 2.2. DAPH

**Figure 5:**
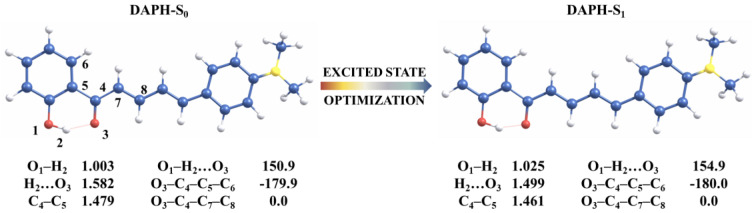
The optimized geometries of DAPH in the S_0_ and S_1_ states. The labeling of atomic color: O: red; C: blue; H: white; N: yellow, the bond length in Å, the angles are in degrees.

**Figure 6:**
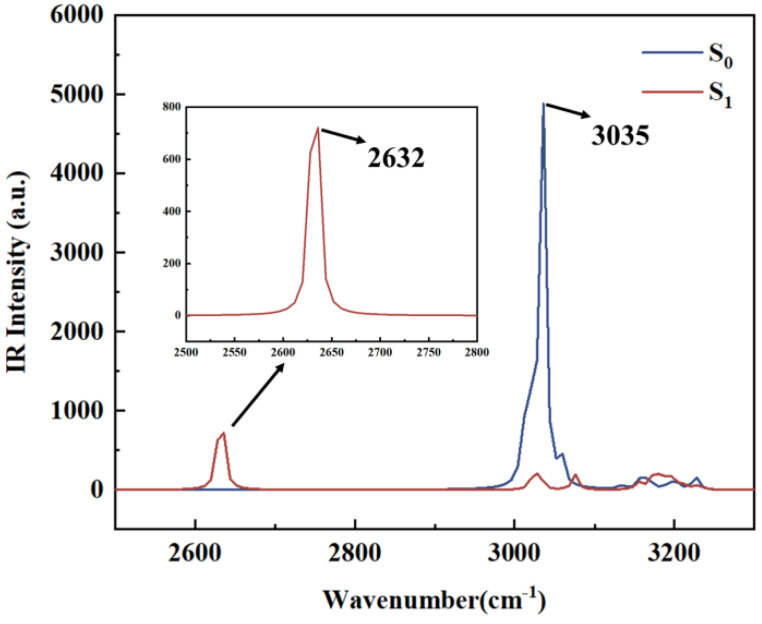
The calculated IR spectra of the DAPH in the spectral region of the O_1_–H_2_ stretching band calculated.

The optimized geometries of DAPH and the corresponding parameters in the S_0_ (S_0_-min) and S_1_ (S_1_-min) states are shown in Figure 5. All structures are ensured to be located at the minimum energy point, without virtual frequency. Compared with the structure in the S_0_ state, the dihedral angles of O_3_–C_4_–C_5_–C_6_ and C_7_–C_8_–C_9_–C_10_ in the S_1_ state change from −179.924° to 179.980° and −0.029° to −0.004°, respectively, indicating that the geometry of DAPH tends to be flattened after photoexcitation. In addition, after the reaction with thiophenol, the DNP group of DAPH-DNP is eliminated, releasing the phenol group, which can form an intramolecular hydrogen bond with the adjacent O atom. The O_1_–H_2_ bond length of DAPH is increased from 1.003 Å (S_0_-min) to 1.025 Å (S_1_-min), and the H_2_…O_3_ bond length is reduced from 1.582 Å (S_0_-min) to 1.499 Å (S_1_-min). Moreover, the bond angles of O_1_–H_2_…O_3_ increased by 4.0° from 150.9° (S_0_-min) to 154.9° (S_1_-min). After photoexcitation, the strength of the hydrogen bond is increased, thereby promoting the ESIPT process. To further verify whether the hydrogen bonding in the S_1_ state is enhanced, the calculated IR vibrational spectra of the relevant hydrogen bond are presented. The results depicted in Figure 6 indicate a significant red-shift of 403 cm^−1^ in the IR vibrational frequency of O_1_–H_2_, from 3035 cm^−1^ (S_0_-min) to 2632 cm^−1^ (S_1_-min), confirming the enhancement of the hydrogen bonding phenomenon. This observation is consistent with the structural analysis view.

**Figure 7:**
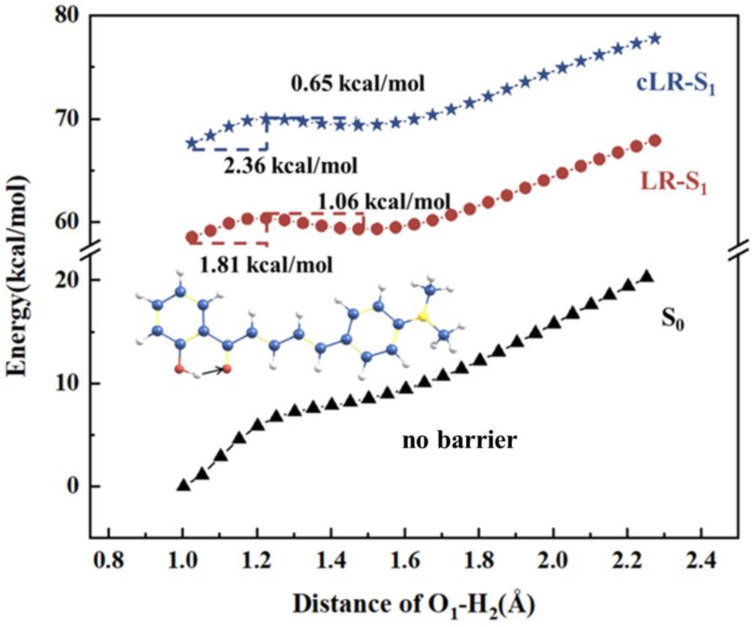
The PECs of the S_0_ and S_1_ states for DAPH along with the O_1_–H_2_ bond length (ωB97XD/TZVP/IEFPCM).

PT process entails a rearrangement of the electron structure within the π system, which subsequently impacts the molecule’s geometry. To gain a deeper understanding of the PT process, we generated PECs for DAPH in both the S_0_ and S_1_ states by conducting a relaxed potential energy scan along the O_1_–H_2_ bond (as illustrated in Figure 7). The results show that in the S_0_ state, the energy of DAPH system continues to rise along the direction of increasing bond length, and there is no low energy point, which means that DAPH-keto cannot exist stably. The PEC of the S_1_ state shows that DAPH-enol can be transformed into DAPH-keto at a low energy point after overcoming an energy barrier of 2.36 kcal/mol at the cLR-ωB97XD/TZVP level. The forward reaction exhibits a higher energy barrier compared to the reverse process (0.65 kcal/mol), suggesting that the conversion of DAPH-keto to DAPH-enol can occur with ease. The above results illustrate that DAPH is not prone to the GSIPT or ESIPT process; this is the reason why DAPH-keto could not be optimized in the S_0_ and S_1_ states. Therefore, the onset of the fluorescence emission of DAPH may not be caused by the ESIPT mechanism proposed in the experiment. It is important to highlight that a relaxed potential energy scan may yield energy profiles that differ significantly from those obtained through a rigid potential energy scan. To verify that the relaxation of molecular geometry does not introduce qualitative changes or alter our conclusions, we also presented the results of a rigid potential energy scan (see Figure S7), which supports the same conclusion.

To study the fluorescence enhancement mechanism, we calculated the absorption and emission spectra of DAPH at the ωB97XD/TZVP level. Moreover, the vertical excitation and emission energies of DAPH involved in orbital transitions, as well as the oscillator strengths, are listed in Table 1. The calculated results show that the S_0_ → S_1_ is the dominant excitation pathway characterized by the transition from HOMO → LUMO, which blocks the occurrence of the PET process. And the excitation energy of DAPH is 2.96 eV (419 nm), which is very consistent with the experiment value (2.68 eV, 462 nm). Compared with DAPH-DNP, the maximum absorption peak of DAPH is shifted in the direction of long wavelength, the 24 nm red-shift indicates that the n-π conjugation effect of the DNP group on the probe is eliminated after the thiolysis reaction. The calculated emission data show that the oscillator strength of DAPH-enol during emission is 1.8406, which means that the decay of DAPH-enol from the S_1_ state to the S_0_ state occurs via radiative transition. In addition, the emission energy of DAPH-enol is also calculated to be 2.74 eV (453 nm), which is significantly overestimated by the ωB97XD functional. This is not surprising because the range-separated functional typically overestimates the energy of the LE state. We then computed the computed emission energy at the LR-B3LYP level, which is 2.14 eV and agrees well with the experimental value (1.90 eV). That is to say, the observed fluorescence should be emitted from DPAH-enol, which agrees with the conclusion we discussed in the PEC section.

**Figure 8:**
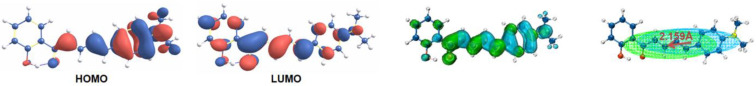
Excitation processes of DAPH. Molecular orbitals are given in blue and red iso-surfaces, holes and electrons are given in blue and green iso-surfaces, respectively (ωB97XD/TZVP/IEFPCM).

**Figure 9:**
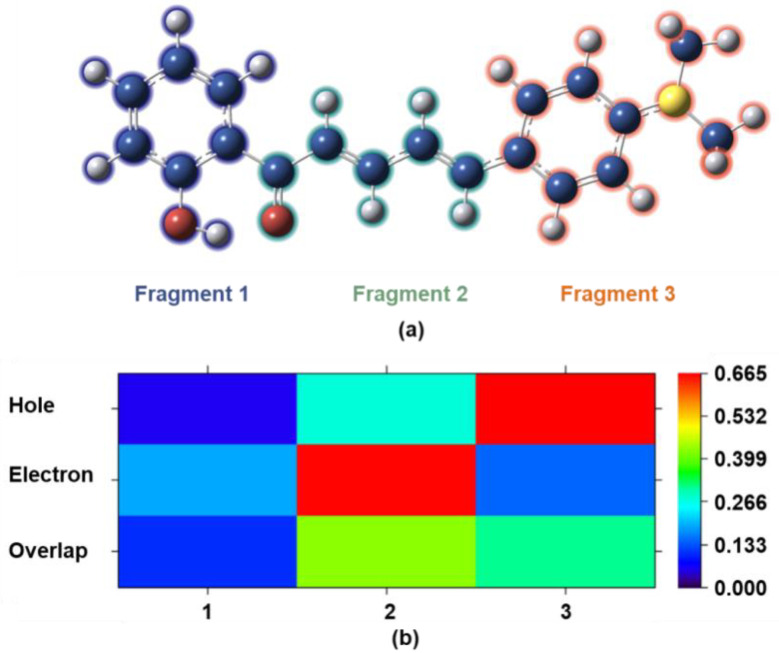
The DAPH molecular fragments electron excitation process analyzed by the IFCT (**a**) and the amount of inter-fragment electron transfer from the S_0_ to S_1_ States (**b**) (ωB97XD/TZVP/IEFPCM).

It is worth mentioning that, according to our conclusion, the significant Stokes shift observed is not attributed to the ESIPT mechanism and requires further investigation to elucidate the underlying factors contributing to the observed phenomenon. As shown in Figure 8, the contribution of the phenol group to HOMO is not obvious, while the contribution to LUMO is significantly increased; the electron distribution of LUMO delocalizes over the DAPH conjugate system after photoexcitation, which indicates that DAPH in the S_1_ state exhibits ICT character. Moreover, to describe the characters of electron excitation more accurately, the hole-electron distribution of DAPH was analyzed. Compared to the hole, the centroid of the electrons shifts in the direction of the phenol group. As listed in Table 2, the D index is 2.159 Å, and the t-index has a negative value (−1.399 Å), significantly less than 0, indicating an obvious separation between holes and electrons. Meanwhile, the IFCT analysis (Figure 9) is provided to describe quantitatively the charge transfer characteristics of DAPH, and is divided into three fragments. Table S4 shows that the contributions of fragment 2 and fragment 3 to electrons and holes are 70.68% and 50.54%, respectively, and the charge transfer differences of fragment 1, fragment 2, and fragment 3 in the excitation of S_0_ → S_1_ are 2.31%, 27.79%, and −30.10%, respectively, indicating that the dominant contribution of electron transfer is from fragment 3 to fragment 2 during excitation. Thus, it further confirms the ICT character of the S_1_ state, and the large Stokes shift observed in the experiment could be caused by the ICT process of DAPH.

**Figure 10:**
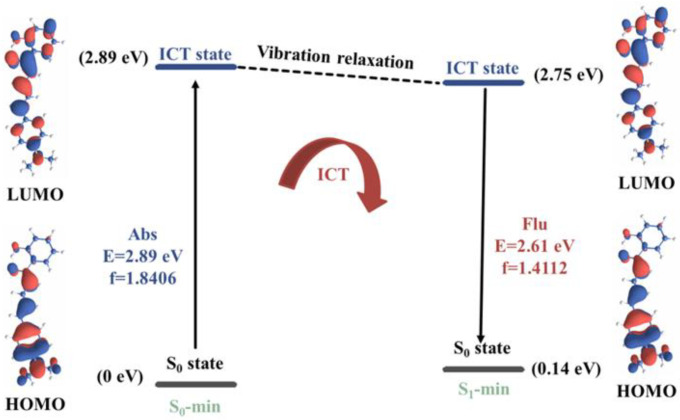
The cLR-ωB97XD/TZVP/IEFPCM calculated energies of DAPH showing the ICT mechanism.

As Figure 10 shows, at the FC point, the S_1_ state is a bright ICT state. By calculating the MOs, the LUMO transformation of DAPH includes intramolecular CT (ICT) from *o*-hydroxyacetophenone to *N*,*N*-dimethylamino. Thus, under photoexcitation, DAPH relaxes directly from the FC point to S_1_-min via vibration relaxation, then returns to the ground state by fluorescence emission.

## 3. Computational Details

Gaussian 16 program package [45] was utilized for all theoretical calculations conducted in this research. The density functional theory (DFT) and time-dependent DFT (TDDFT) approaches were employed with B3LYP functional and TZVP [46,47] basis set to optimize the geometry. The range-separated functional ωB97XD approach was used to calculate the single point energy. Furthermore, to make the calculation results more accurate, the polarizable continuum model using the integral equation formalism variant (IEFPCM) [34,48] was utilized with water (H_2_O, ε = 78.3553) as the solvent. To verify the TDDFT results, we also used the COSMO-ADC(2)/PTED method to compute the excitation energies. For comparison, all the results by B3LYP functional are also provided in the Supplementary Materials (Tables S5–S8, Figures S1, S2 and S5–S7).

All the geometric structures were at local minima with no imaginary frequency through harmonic frequency analysis. In order to analyze the ESIPT process, the relaxed potential energy curves (PECs) were constructed for both S_0_ and S_1_ states by fixing the O–H distance and incrementally varying it by 0.05 Å. The fluorescence quenching mechanism of the probe was investigated by analyzing the FMOs and the fragment charge transfer via Multiwfn 3.8 software [33].

To investigate the charge transfer mechanism, we utilized hole-electron analysis. The methodology involved using various indices, including the centroid distance (*D*) index, the degree of overlap (*Sr*) index, the width distribution (*H*) index, degree of separation (*t*) index, hole delocalization index (*HDI*), and electron delocalization index (*EDI*) via the Multiwfn program. The Sr index was utilized to measure the extent of hole-electron overlap, while the *D* index quantified the distance between the hole and the electron’s mass center. The *H* index provided an average distribution range of electrons and holes, and the t index evaluated the separation of electrons and holes. *HDI* and *EDI* were used to assess the degree of hole-electron delocalization, which indicated the uniformity of the distribution. The relevant formula is as follows:

$$S_{r}\;index=\int S_{r}(r)dr=\int \sqrt{\rho^{hole}(r)\rho^{ele}(r)dr}$$

$$D_{x}=\left|X_{ele}-X_{hole}\right|\;D_{y}=\left|Y_{ele}-Y_{hole}\right|\;D_{Z}=|Z_{ele}-Z_{hole}|$$

$$D\;index=\sqrt{{{(D}_{x})}^{2}+{\left(D_{y}\right)}^{2}+({D_{z})}^{2}}$$

$$H\;index=(\left|\sigma_{ele}\right|+\left|\sigma_{hole}\right|)/2$$

$$t\;index=D\;index-H_{CT}$$

$$HDI=100\times \sqrt{\int [{\rho^{hole}(r)]}^{2}dr}$$

$$EDI=100\times \sqrt{\int {\left[\rho^{ele}\left(r\right)\right]}^{2}dr}$$

## 4. Conclusions

The work provided a detailed theoretical basis for the photophysical characteristics of DAPH-DNP and DAPH based on the DFT and TD-DFT methods at the ωB97XD/TZVP level. The fluorescence quenching pathway of DAPH-DNP is caused by the typical d-PET mechanism. By comparing the charge transfer characteristics of the S_0_, S_1_, and S_2_ states of DAPH-DNP using hole-electron and IFCT analysis methods, it was found that the DNP group makes a significant contribution to the charge transfer excitation. The geometric structure information and infrared vibration spectra data show that the intramolecular hydrogen bonds of DAPH are enhanced in the S_1_ state. The calculated PECs indicate that the ESIPT process could not occur in DAPH due to the unstable nature of the keto structure; it quickly reverted back to the enol structure. ICT is the main cause of the large Stokes shift, which is inconsistent with the conclusion proposed in the experiment. This further suggests that the fluorescence enhancement of DAPH is not based on the ESIPT process. By the analysis of the FMOs of DAPH, the process of electronic excitation was found to be accompanied by the ICT process, indicating that the large Stokes shift observed in the experiment can be attributed to the ICT characteristic in the S_1_ state of DAPH-enol. Our theoretical study not only explains the sensing mechanism but also confirms the inefficiency of the ESIPT process in the probe DAPH-DNP, which is of great significance for the synthesis of novel probes in the future.

## Figures and Tables

**Table 1:** Detailed theoretical and experimental spectral data for DAPH-DNP and DPAH (ωB97XD/TZVP/IEFPCM).

	Electronic Transition	Energy (nm/eV)		f	Contrib	CI	Exp (nm/eV)
		LR	cLR				
**DAPH-** **DNP**							
Absorption	S_0_ → S_1_	395/3.14	395/3.14	1.8031	H → L + 2	81.3%	447/2.77
Absorption	S_0_ → S_2_	335/3.70	339/3.66	0.0007	H-4 → L + 2	48.1%	/
Emission	S_1_ → S_0_	800/1.55	800/1.55	0.4340	H → L	63.8%	/
Emission	S_2_ → S_0_	477/2.60	458/2.71	1.8673	H → L + 2	70.1%	/
**DAPH**							
Absorption	S_0_ → S_1_	419/2.96	429/2.89	1.8406	H → L	86.1%	462/2.68
Emission	S_1_ → S_0_	453/2.74	475/2.61	1.8491	H → L	88.1%	654/1.90

**Table 2:** The calculated results of the excited states for DAPH-DNP and DAPH, including the centroid distance (D), the degree of overlap (Sr), the width distribution (H), degree of separation (t), hole delocalization index (HDI), and electron delocalization index (EDI) (ωB97XD/TZVP/IEFPCM).

	D (Å)	Sr	H (Å)	t (Å)	HDI	EDI
**DAPH-DNP**						
S_0_ → S_1_	2.979	0.62145	3.658	−0.122	6.88	7.21
S_0_ → S_2_	2.225	0.38421	2.693	0.201	17.99	6.99
S_1_ → S_0_	3.671	0.57021	3.971	0.192	6.84	5.99
**DAPH**						
S_0_ → S_1_	2.159	0.73058	3.814	−1.399	6.97	7.30
S_1_ → S_0_	1.991	0.73102	3.868	−1.599	6.91	7.30

## Data Availability

The authors confirm that the data supporting the findings of this study are available within the article [and/or] its Supplementary Materials.

## Supplementary Materials

The following supporting information can be downloaded at: https://www.mdpi.com/article/10.3390/molecules28196921/s1, Table S1: Comparing calculated spectral data for DAPH based on different functionals (B3LYP, CAM-B3LYP, PBE0, and PBEPBE); Table S2: Contribution of different segments of DAPH-DNP to the electron orbitals from S_0_ to S_1_ state and S_3_ state (ωB97XD/TZVP/IEFPCM); Table S3. The COSMO-ADC(2)/PTED computed vertical excitation energies (in eV) of DAPH-DAP. Table S4: Contribution of different segments of DAPH to the electron orbitals from S_0_ to S_1_ state (ωB97XD/TZVP/IEFPCM); Table S5: Detailed theoretical and experimental spectral data for DAPH-DNP and DPAH (B3LYP/TZVP/IEFPCM); Table S6: The calculated results of the excited states for DAPH-DNP and DAPH, including the centroid distance (D), the degree of overlap (Sr), the width distribution (H), degree of separation (t), hole delocalization index (HDI), and electron delocalization index (EDI) (B3LYP/TZVP/IEFPCM); Table S7: Contribution of different segments of DAPH-DNP to the electron orbitals from S_0_ to S_1_ state and S_3_ state (B3LYP/TZVP/IEFPCM); Table S8. Contribution of different segments of DAPH to the electron orbitals from S_0_ to S_1_ state (B3LYP/TZVP/IEFPCM); Figure S1: The B3LYP/TZVP/IEFPCM calculated energies of DAPH-DNP showing the PET mechanism; Figure S2: Excitation processes of DAPH-DNP (molecular orbitals are given in blue and red iso-surfaces, holes and electrons are given in blue and green iso-surfaces, respectively); Figure S3: The IFCT analyzing the electron excitation process of DAPH-DNP molecular fragments (a), the amount of electron transfer between fragments from the S_0_ to S_1_ (b) and S_3_ states (c); Figure S4: Frontier molecular orbitals showing the excitation process of DAPH; Figure S5: The B3LYP/TZVP/IEFPCM calculated energies of DAPH showing the ICT mechanism; Figure S6: The PECs of the S_0_ and S_1_ states for DAPH along with the O_1_–H_2_ bond length; Figure S7: The PECs of the S_0_ and S_1_ states for DAPH along with the O_1_–H_2_ bond length (rigid scan).
