# Supplementary material for: Theoretical Investigation on the “ON-OFF” Mechanism of a Fluorescent Probe for Thiophenols: Photoinduced Electron Transfer and Intramolecular Charge Transfer
Source: Molecules. 2023 Oct 3;28(19):6921. doi: 10.3390/molecules28196921 (PMC10574459; doi:10.3390/molecules28196921)
Supplement: Supplementary file 1 [file molecules-28-06921-s001.zip › molecules-2587533-supplementary.pdf]

Table S1. Comparing calculated spectral data for DAPH based on different functionals (B3LYP, CAM-B3LYP, PBE0, and PBEPBE).

		Energy (nm/eV)	f	Contrib	CI
**B3LYP**	Abs.	498/2.49	1.4967	H→L	99.2%
	Flu.	579/2.14	1.7921	H→L	99.0%
**CAM-B3LYP**	Abs.	410/3.02	1.7215	H→L	88.7%
	Flu.	552/2.25	2.0580	H→L	94.7%
**PBE0**	Abs.	478/2.59	1.5612	H→L	98.9%
	Flu.	565/2.19	1.9062	H→L	98.5%
**PBEPBE**	Abs.	579/2.14	1.2802	H→L	94.7%
	Flu.	697/1.78	1.2523	H→L	98.4%

Table S2. Contribution of different segments of DAPH-DNP to the electron orbitals from S_0 to S_1 state and S_2 state (ωB97XD/TZVP/IEFPCM).

Fragment	Hole(%)	Electron(%)	Overlap(%)	Difference(%)
**S_1**				
1	21.63	34.48	27.31	12.84
2	78.37	65.52	71.66	-12.84
**S_2**				
1	96.87	67.32	80.76	-29.55
2	3.13	32.68	10.11	29.55

Table S3. The COSMO-ADC(2)/PTED computed vertical excitation energies (in eV) of DAPH-DAP.

	S_0-min	LE-min	CT-min
**LE**	2.54	1.85	1.18
**CT**	3.00	2.40	0.08

Table S4. Contribution of different segments of DAPH to the electron orbitals from S_0 to S_1 state (ωB97XD/TZVP/IEFPCM).

Fragment	Hole(%)	Electron(%)	Overlap(%)	Difference(%)
1	6.56	8.87	7.63	2.31
2	42.90	70.68	55.06	27.79
3	50.54	20.45	32.15	-30.10

Table S5. Detailed theoretical and experimental spectral data for DAPH-DNP and DPAH (B3LYP/TZVP/IEFPCM).

	Electronic transition	Energy (nm/eV)	f	Contrib	CI	Exp (nm/eV)
**DAPH-DNP**						
Absorption	S_0→S_1	711/1.74	0.0002	H→L	89.6%	/
Absorption	S_0→S_2	648/1.91	0.0145	H→L+1	89.7%	/
Absorption	S_0→S_3	473/2.62	1.4757	H→L+2	98.5%	447/2.77
Emission	S_1→S_0	1468/0.84	0.0044	L→H	99.5%	/
Emission	S_2→S_0	1468/0.84	0.0044	L→H	99.5%	/
**DAPH**						
Absorption	S_0→S_1	497/2.49	1.4867	H→L	99.3%	462/2.68
Emission	S_1→S_0	581/2.14	1.7921	H→L	99.0%	654/1.90

Table S6. The calculated results of the excited states for DAPH-DNP and DAPH, including the centroid distance (D), the degree of overlap (Sr), the width distribution (H), degree of separation (t), hole delocalization index (HDI), and electron delocalization index (EDI) (B3LYP/TZVP/IEFPCM).

	D (Å)	Sr	H (Å)	t (Å)	HDI	EDI
**DAPH-DNP**						
$S_0 \rightarrow S_1$	9.718	0.04367	3.244	7.112	8.16	11.96
$S_0 \rightarrow S_2$	11.644	0.07328	3.282	8.945	8.29	12.53
$S_0 \rightarrow S_3$	4.194	0.62668	3.924	0.842	8.45	7.42
**DAPH**						
$S_0 \rightarrow S_1$	4.332	0.61323	4.031	0.547	8.11	7.66

Table S7. Contribution of different segments of DAPH-DNP to the electron orbitals from S_0 to S_1 state and S_3 state (B3LYP/TZVP/IEFPCM).

Fragment	Hole (%)	Electron (%)	Overlap (%)	Difference (%)
**S_1**				
1	0.08	97.09	2.73	97.01
2	1.01	3.08	1.76	2.07
3	35.20	-0.17	0.00	-35.37
4	63.72	0.00	0.22	-63.72
**S_3**				
1	0.19	1.79	0.58	1.61
2	2.18	12.56	5.24	10.38
3	28.40	69.27	44.36	40.86
4	69.22	16.38	33.67	-52.85

Table S8. Contribution of different segments of DAPH to the electron orbitals from S_0 to S_1 state (B3LYP/TZVP/IEFPCM).

Fragment	Hole (%)	Electron (%)	Overlap (%)	Difference (%)
1	5.90	19.16	10.63	13.27
2	27.64	66.19	42.78	38.55
3	66.46	14.64	31.20	-51.82

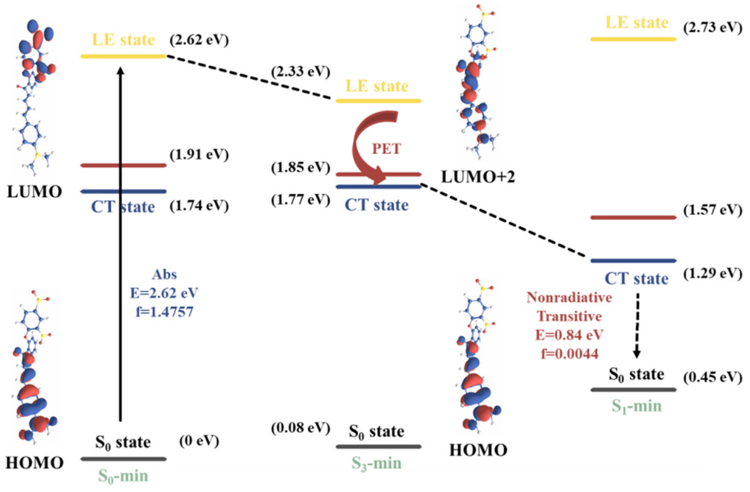

Figure S1. The B3LYP /TZVP/IEFPCM calculated energies of DAPH-DNP showing the PET mechanism.

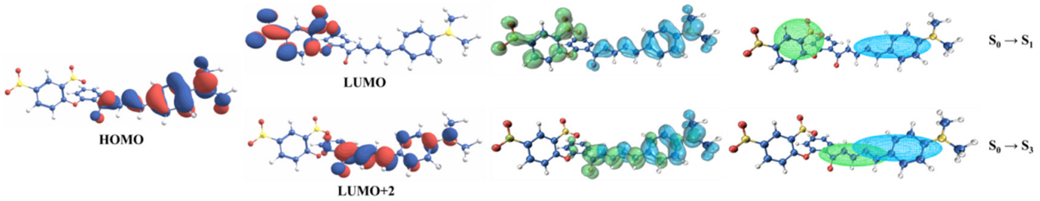

Figure S2. Excitation processes of DAPH-DNP (molecular orbitals are given in blue and red iso-surfaces, holes and electrons are given in blue and green iso-surfaces, respectively).

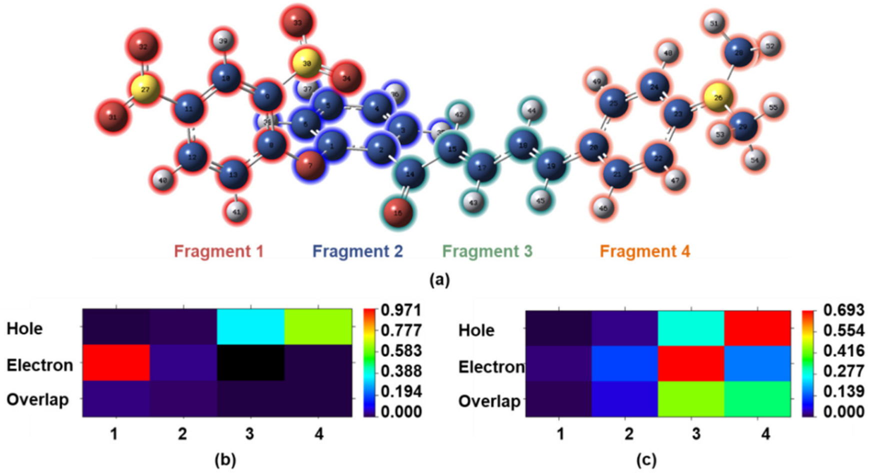

Figure S3. The IFCT analyzing the electron excitation process of DAPH-DNP molecular fragments (a), the amount of electron transfer between fragments from the S_0 to S_1 (b) and S_3 states (c).

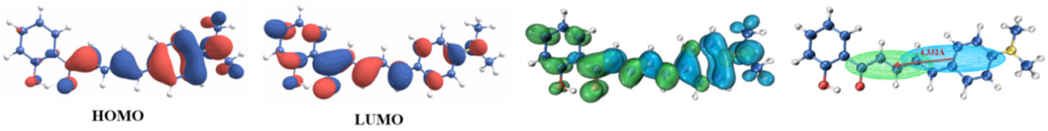

Figure S4. Frontier molecular orbitals showing the excitation process of DAPH.

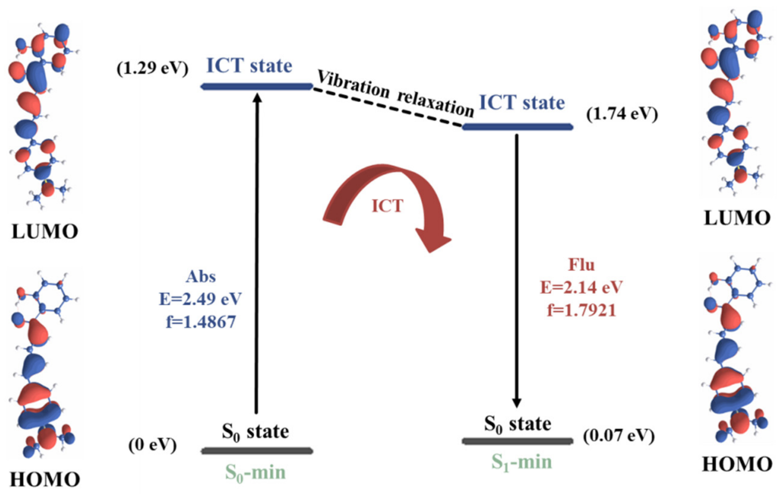

Figure S5. The B3LYP/TZVP/IEFPCM calculated energies of DAPH showing the ICT mechanism.

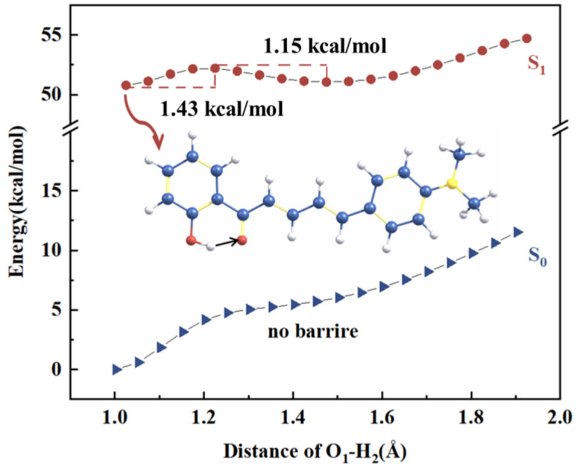

Figure S6. The PECs of the S_0 and S_1 states for DAPH along with the O_1–H_2 bond length.

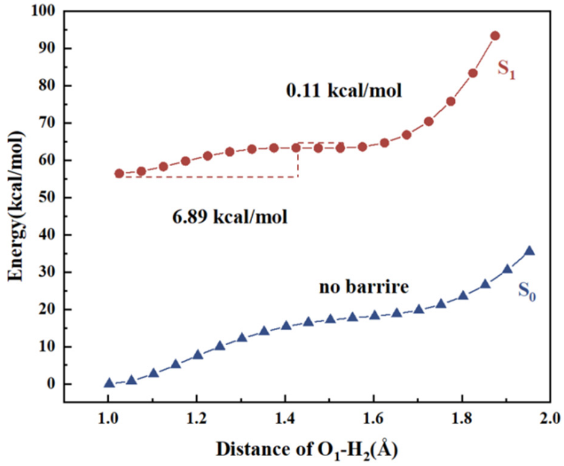

Figure S7. The PECs of the S_0 and S_1 states for DAPH along with the O_1–H_2 bond length (rigid scan).

B3LYP-D3(BJ)/TZVP/IEFPCM optimized geometries

DAPH-DNP **S_0-min**

Atom	x	y	z
C1	-2.944084	1.935293	-0.328902
C2	-1.634332	2.419932	-0.299744
C3	-1.366685	3.520396	0.517650
C4	-2.368727	4.130162	1.262089
C5	-3.664125	3.629108	1.210933
C6	-3.954978	2.525908	0.416329
C7	-3.219975	0.845960	-1.149620
C8	-4.134089	-0.079270	-0.777886
C9	-4.184879	-0.704414	0.481019
C10	-5.172700	-1.624445	0.785695
C11	-6.087169	-1.961691	-0.194697
C12	-6.042861	-1.393203	-1.463702
C13	-5.071847	-0.451614	-1.741738
C14	-0.538484	1.802499	-1.120097
C15	0.718066	1.554911	-0.429788
C16	-0.726897	1.543726	-2.310784
C17	1.794045	1.024944	-1.066713
C18	3.044614	0.729577	-0.457448
C19	4.079656	0.191095	-1.156105
C20	5.389539	-0.154192	-0.672358
C21	6.327559	-0.722129	-1.555260
C22	7.598037	-1.074814	-1.153002
C23	8.015099	-0.876617	0.185891
C24	7.074424	-0.299645	1.079582
C25	5.810584	0.044268	0.658220
N26	9.264959	-1.224183	0.601144
N27	-7.127879	-2.946665	0.120334
C28	9.683038	-0.972812	1.972354
C29	10.221472	-1.783996	-0.341674
N30	-3.170169	-0.463129	1.515491
O31	-7.923644	-3.241929	-0.764298
O32	-7.148726	-3.422495	1.250361
O33	-3.534685	-0.484324	2.684283
O34	-2.015784	-0.287658	1.149658
H35	-0.357384	3.908860	0.556980
H36	-2.138691	4.988961	1.878288
H37	-4.455261	4.093989	1.784251
H38	-4.962709	2.135041	0.371161
H39	-5.206992	-2.083874	1.761060
H40	-6.770361	-1.674763	-2.209578

H41	-5.029049	0.034628	-2.705772
H42	0.770509	1.779919	0.627923
H43	1.691760	0.805048	-2.126366
H44	3.149453	0.949637	0.599853
H45	3.910213	-0.010044	-2.211204
H46	6.041573	-0.887628	-2.587634
H47	8.274324	-1.504946	-1.875678
H48	7.344992	-0.122751	2.109354
H49	5.131766	0.481181	1.379230
H50	9.663939	0.094830	2.211296
H51	9.043413	-1.497074	2.687298
H52	10.699383	-1.332087	2.102234
H53	9.848023	-2.711031	-0.784878
H54	10.448676	-1.083162	-1.150896
H55	11.144708	-2.008629	0.183610

DAPH-DNP **S_1-min**

Atom	x	y	z
C1	-2.988177	2.005253	-0.120007
C2	-1.730915	2.621412	-0.099879
C3	-1.485503	3.611733	0.853736
C4	-2.468956	4.003000	1.752546
C5	-3.706604	3.368970	1.727165
C6	-3.965556	2.366246	0.799856
C7	-3.221357	1.043024	-1.073790
C8	-4.064228	0.001991	-0.756681
C9	-3.793928	-0.909086	0.292597
C10	-4.747393	-1.893043	0.561133
C11	-5.882434	-1.991285	-0.225431
C12	-6.130682	-1.135846	-1.293725
C13	-5.203870	-0.132891	-1.536013
C14	-0.653135	2.190436	-1.032626
C15	0.661318	1.916400	-0.417757
C16	-0.841880	2.044155	-2.233695
C17	1.676466	1.362454	-1.126175
C18	2.915804	1.008284	-0.540541
C19	3.918258	0.399082	-1.255635
C20	5.174700	-0.013770	-0.754279
C21	6.091777	-0.653401	-1.634483
C22	7.314413	-1.081150	-1.213593
C23	7.718766	-0.894601	0.145381
C24	6.800964	-0.249642	1.036094
C25	5.582996	0.169192	0.598443
N26	8.917137	-1.309559	0.572501

N27	-6.850251	-3.049366	0.076924
C28	9.332287	-1.114271	1.964934
C29	9.853028	-1.974593	-0.339153
N30	-2.623376	-0.876163	1.077091
O31	-7.819329	-3.179254	-0.668272
O32	-6.657158	-3.760826	1.060302
O33	-2.642642	-1.465252	2.228742
O34	-1.536937	-0.388326	0.580979
H35	-0.513536	4.087843	0.883828
H36	-2.266658	4.783587	2.473505
H37	-4.477564	3.650324	2.432913
H38	-4.924550	1.866928	0.786132
H39	-4.569426	-2.577781	1.372716
H40	-7.023634	-1.234486	-1.888989
H41	-5.365871	0.588186	-2.325200
H42	0.780409	2.106747	0.640364
H43	1.522643	1.157826	-2.180681
H44	3.047998	1.224063	0.512734
H45	3.734205	0.203143	-2.306716
H46	5.802551	-0.803314	-2.666251
H47	7.976556	-1.562670	-1.914623
H48	7.071627	-0.091941	2.067469
H49	4.919149	0.650326	1.301831
H50	9.351919	-0.053068	2.212717
H51	8.653649	-1.631739	2.642900
H52	10.328717	-1.520609	2.090841
H53	9.416049	-2.892546	-0.732078
H54	10.114927	-1.315505	-1.166795
H55	10.754306	-2.223113	0.208197

DAPH-DNP **S_2-min**

Atom	x	y	z
C1	-2.988270	2.005340	-0.120032
C2	-1.730970	2.621401	-0.099935
C3	-1.485459	3.611646	0.853738
C4	-2.468817	4.002892	1.752661
C5	-3.706498	3.368928	1.727332
C6	-3.965563	2.366310	0.799934
C7	-3.221651	1.043273	-1.073948
C8	-4.064297	0.002079	-0.756735
C9	-3.793790	-0.908997	0.292493
C10	-4.747154	-1.893005	0.561118
C11	-5.882317	-1.991377	-0.225303
C12	-6.130719	-1.135939	-1.293563

C13	-5.204008	-0.132918	-1.535950
C14	-0.653285	2.190342	-1.032724
C15	0.661232	1.916431	-0.417928
C16	-0.842116	2.043943	-2.233766
C17	1.676313	1.362355	-1.126347
C18	2.915686	1.008266	-0.540729
C19	3.918095	0.398882	-1.255725
C20	5.174550	-0.013924	-0.754346
C21	6.091546	-0.653795	-1.634458
C22	7.314198	-1.081499	-1.213563
C23	7.718676	-0.894648	0.145345
C24	6.800952	-0.249452	1.035956
C25	5.582969	0.169333	0.598298
N26	8.917067	-1.309433	0.572589
N27	-6.849991	-3.049457	0.077196
C28	9.332099	-1.113671	1.964994
C29	9.853129	-1.974612	-0.338809
N30	-2.623132	-0.876047	1.076835
O31	-7.819376	-3.179235	-0.667621
O32	-6.656584	-3.761113	1.060378
O33	-2.642237	-1.465185	2.228464
O34	-1.536808	-0.388041	0.580650
H35	-0.513481	4.087735	0.883799
H36	-2.266418	4.783434	2.473638
H37	-4.477392	3.650260	2.433160
H38	-4.924578	1.867034	0.786229
H39	-4.569020	-2.577744	1.372663
H40	-7.023726	-1.234636	-1.888737
H41	-5.366153	0.588152	-2.325109
H42	0.780462	2.106963	0.640143
H43	1.522416	1.157560	-2.180808
H44	3.047930	1.224264	0.512497
H45	3.733992	0.202739	-2.306762
H46	5.802235	-0.803932	-2.666172
H47	7.976254	-1.563192	-1.914547
H48	7.071730	-0.091492	2.067267
H49	4.919210	0.650671	1.301629
H50	9.352115	-0.052353	2.212278
H51	8.653146	-1.630549	2.643086
H52	10.328340	-1.520372	2.091227
H53	9.416245	-2.892640	-0.731653
H54	10.115184	-1.315659	-1.166509
H55	10.754305	-2.223029	0.208757

DAPH-DNP **S_3-min**

Atom	x	y	z
C1	-2.830816	1.812764	-0.440837
C2	-1.471280	2.174789	-0.458545
C3	-1.119764	3.308406	0.296922
C4	-2.055847	4.040058	1.010991
C5	-3.395423	3.661342	0.989604
C6	-3.780452	2.542908	0.256596
C7	-3.221966	0.690876	-1.170994
C8	-4.231073	-0.097185	-0.746646
C9	-4.388151	-0.595056	0.560958
C10	-5.475762	-1.379721	0.904941
C11	-6.388487	-1.717387	-0.076767
C12	-6.243496	-1.277329	-1.389591
C13	-5.173059	-0.467578	-1.709960
C14	-0.453811	1.433962	-1.244670
C15	0.866758	1.322352	-0.662954
C16	-0.735224	0.921907	-2.356747
C17	1.899249	0.648759	-1.278210
C18	3.181315	0.489879	-0.701022
C19	4.226593	-0.184658	-1.294415
C20	5.534557	-0.365051	-0.740855
C21	6.510871	-1.091168	-1.472010
C22	7.779187	-1.299124	-0.993700
C23	8.171153	-0.785980	0.274924
C24	7.200081	-0.055259	1.016296
C25	5.935963	0.143855	0.524421
N26	9.423001	-0.985833	0.759639
N27	-7.530382	-2.560993	0.281179
C28	9.805346	-0.464049	2.065522
C29	10.408929	-1.733168	-0.011003
N30	-3.393189	-0.359107	1.613232
O31	-8.319431	-2.868085	-0.608402
O32	-7.642329	-2.918068	1.451300
O33	-3.797919	-0.245927	2.764624
O34	-2.215540	-0.319311	1.284166
H35	-0.087643	3.631592	0.296246
H36	-1.744055	4.911182	1.572363
H37	-4.140446	4.230571	1.529291
H38	-4.819461	2.241608	0.230286
H39	-5.588489	-1.738777	1.915749
H40	-6.970849	-1.554918	-2.137095
H41	-5.049445	-0.080965	-2.711414
H42	1.038359	1.754572	0.315637
H43	1.714908	0.213321	-2.254940

H44	3.329571	0.938026	0.276833
H45	4.055895	-0.626289	-2.271113
H46	6.241202	-1.494389	-2.440172
H47	8.479489	-1.860409	-1.592728
H48	7.453260	0.353209	1.982381
H49	5.234751	0.705274	1.125768
H50	9.720283	0.625057	2.092264
H51	9.175354	-0.882673	2.854197
H52	10.836097	-0.735560	2.265035
H53	10.074311	-2.758660	-0.186452
H54	10.594643	-1.256291	-0.976230
H55	11.340051	-1.763254	0.544218

DAPH **S_0-min**

Atom	x	y	z
C1	5.964294	-0.476350	-0.000890
C2	4.700472	0.171764	0.000436
C3	4.684522	1.578605	0.002303
C4	5.851202	2.318642	0.002666
C5	7.084337	1.659642	0.001116
C6	7.141644	0.278384	-0.000648
O7	6.073459	-1.817055	-0.002373
C8	3.468819	-0.646355	-0.000127
O9	3.568608	-1.901451	-0.000375
C10	2.168049	-0.010950	-0.000559
C11	1.019384	-0.738508	-0.000377
C12	-0.291577	-0.190262	-0.000812
C13	-1.403261	-0.974952	-0.000003
C14	-2.779115	-0.558619	-0.000136
C15	-3.798141	-1.530505	0.001492
C16	-5.135045	-1.195649	0.001626
C17	-5.541842	0.161234	0.000103
C18	-4.518159	1.145713	-0.001733
C19	-3.189298	0.790383	-0.001765
N20	-6.857938	0.509754	0.000404
C21	-7.249844	1.911520	-0.002922
C22	-7.888928	-0.517233	0.002936
H23	3.741020	2.103678	0.003610
H24	5.808624	3.399411	0.004168
H25	8.003664	2.231789	0.001357
H26	8.088795	-0.245183	-0.001749
H27	5.130122	-2.156761	-0.001774
H28	2.104780	1.067013	-0.001084
H29	1.107229	-1.821216	0.000200

H30	-0.381852	0.890992	-0.001696
H31	-1.246939	-2.051003	0.000948
H32	-3.521726	-2.578607	0.002649
H33	-5.871481	-1.984489	0.002797
H34	-4.776103	2.193729	-0.002992
H35	-2.448516	1.579481	-0.003126
H36	-6.877320	2.430215	-0.890777
H37	-8.333710	1.974473	-0.003546
H38	-6.878035	2.434289	0.882853
H39	-7.818556	-1.153491	0.889789
H40	-8.863664	-0.039139	0.004869
H41	-7.822377	-1.154428	-0.883587

DAPH **S_1-min**

Atom	x	y	z
C1	-5.955057	-0.381417	0.000343
C2	-4.644698	0.193788	-0.000182
C3	-4.562591	1.604291	-0.000625
C4	-5.691009	2.401438	-0.000450
C5	-6.961914	1.814264	0.000139
C6	-7.088173	0.433411	0.000531
O7	-6.115393	-1.716699	0.000689
C8	-3.484437	-0.694490	-0.000238
O9	-3.663347	-1.972006	-0.000218
C10	-2.151083	-0.162678	-0.000183
C11	-1.023371	-0.953947	-0.000221
C12	0.290409	-0.426069	-0.000129
C13	1.436918	-1.187429	-0.000114
C14	2.780072	-0.688880	-0.000048
C15	3.865946	-1.601750	0.000046
C16	5.172932	-1.185036	0.000070
C17	5.493949	0.201463	0.000006
C18	4.411310	1.125886	-0.000023
C19	3.110466	0.692809	-0.000067
N20	6.782626	0.626318	-0.000029
C21	7.095478	2.049851	0.000905
C22	7.881141	-0.331872	-0.000482
H23	-3.593765	2.082687	-0.001149
H24	-5.589591	3.478998	-0.000808
H25	-7.848743	2.435391	0.000283
H26	-8.061577	-0.040832	0.000955
H27	-5.157892	-2.082613	0.000382
H28	-2.017229	0.910742	-0.000068
H29	-1.147849	-2.031356	-0.000326

H30	0.375797	0.656401	-0.000063
H31	1.328469	-2.267478	-0.000154
H32	3.652804	-2.663556	0.000134
H33	5.958845	-1.924391	0.000249
H34	4.607123	2.186956	-0.000104
H35	2.323912	1.434523	-0.000148
H36	6.688204	2.540278	0.888409
H37	8.172865	2.173273	0.001590
H38	6.689218	2.541255	-0.886538
H39	7.845266	-0.968919	-0.887578
H40	8.820723	0.209880	-0.001962
H41	7.847245	-0.967632	0.887657
